# Creation, evolution, and dissolution of social groups

**DOI:** 10.1038/s41598-021-96805-7

**Published:** 2021-09-01

**Authors:** James Flamino, Boleslaw K. Szymanski, Ashwin Bahulkar, Kevin Chan, Omar Lizardo

**Affiliations:** 1 Network Science and Technology Center, Rensselaer Polytechnic Institute, Troy, NY 12180 USA; 2 Department of Physics, Applied Physics, and Astronomy, Rensselaer Polytechnic Institute, Troy, NY 12180 USA; 3 Department of Computer Science, Rensselaer Polytechnic Institute, Troy, NY 12180 USA; 4 Społeczna Akademia Nauk, Łódź, Poland; 5 U.S. Army Research Laboratory, Adelphi, MD 20783 USA; 6 Department of Sociology, University of California, Los Angeles, CA 90095 USA

**Keywords:** Complex networks, Computational science, Human behaviour, Computer science

## Abstract

Understanding why people join, stay, or leave social groups is a central question in the social sciences, including computational social systems, while modeling these processes is a challenge in complex networks. Yet, the current empirical studies rarely focus on group dynamics for lack of data relating opinions to group membership. In the NetSense data, we find hundreds of face-to-face groups whose members make thousands of changes of memberships and opinions. We also observe two trends: opinion homogeneity grows over time, and individuals holding unpopular opinions frequently change groups. These observations and data provide us with the basis on which we model the underlying dynamics of human behavior. We formally define the utility that members gain from ingroup interactions as a function of the levels of homophily of opinions of group members with opinions of a given individual in this group. We demonstrate that so-defined utility applied to our empirical data increases after each observed change. We then introduce an analytical model and show that it accurately recreates the trends observed in the NetSense data.

## Introduction

Most social interactions among humans occur in the context of a set of relatively small face-to-face groups, and not in isolated dyads^1–6^. In addition, these groups are not static. Instead, their membership changes over time with individuals constantly deciding whether to stay in their current groups and perhaps adjust some opinions, or leave to join other groups^7^. To date, however, the dynamics of human group formation, evolution, and dissolution in real social groups remain poorly understood^8,9^. Among humans, groups represent a level of association mediating between the individual and whole societies, producing a variety of personal benefits, including companionship and support^1^, while also allowing people to come together to pursue instrumental goals or work together toward common purposes^10^. Despite the introduction of telecommunication as an entirely different medium of interaction between individuals, the importance of offline groups as the primary nexus of social life has prevailed. Indeed, new technologically mediated interactions such as texting, online chatting, and social media complement rather than replace face-to-face interaction in small groups, and a great majority of online ties ultimately relies on (or emerges from) offline face-to-face interactions^11^. Extended to social connections within face-to-face groups, computational and empirical literature have found that these groups are continually evolving rather than being static^5,8,9,12,13^. Such evolution has been aided by the tendency of the contemporary societies to relax social and institutional restrictions on group joining and leaving behavior compared to the past or to the societies that are more traditional^10,14^.

The previous theoretical works identified homophilic interactions within groups and communities as the primary component of the membership benefits^4,7^. Recent papers^15–17^ separately consider a role of homophily in popularity, ranking of minorities, and structural change in social networks. Here, we formalize a notion of utility of group membership as a function of compatibility of opinions between a member and all others in its group. The utility enables us to integrate in a single model the three aforementioned phenomena and to account for the two dynamic patterns observed in empirical data. We find that changes of opinions or group memberships that increase members’ utility recreate the group dynamic patterns observed in empirical data and the theoretically postulated role of homophily in these dynamics. This agreement validates our utility maximization hypothesis. Subsequently, we introduce a predictive model based on utility maximization. This model performs well on forecasting opinions and group membership dynamics within the data, which further validates our approach.

In this paper we assume a broad definition of “groups” since within the social sciences, there are a variety of ways of defining groups^1,3,18^. Some definitions emphasize enduring affective ties among members, while others point to a unique sociometric signature (e.g., fully connected cliques). However, all approaches point to three features that seem to be characteristic of all human groups^18^. First, groups are located in specific settings allowing for face-to-face interaction within relatively small spatial distances (dormitories, homes, workplaces), happening at specific times (mornings, evenings)^3,7^. Second, groups are relatively small^1^. After a human gathering reaches a limit size, it loses its capacity to function as a coordinated group, and becomes a “crowd” or a “mass” instead^10^. Finally, groups can overlap. This means that persons are not constrained to belong to only one group. Instead, groups share members, and people need to make decisions (given a limited budget) as to how much time and energy they will dedicate to each group.

These three features, namely, specific location in time and space, relatively small size, and possibility of overlap, inform our approach to group detection and to analysis of group composition dynamics presented here. We are agnostic as to whether “strong” ties exist among group members as we define them; instead, we allow for people to be either weakly or strongly attached to the groups to which they belong as that becomes an endogenous factor driving the dynamics of change in group membership we observe. By definition, since, as in previous work^8,19–21^, we use data sensitive to spatial co-location to define groups, every group we observe is a sociometric clique^3^.

## Results

### Data

In this paper, we use the NetSense study that follows 196 randomly selected students from the incoming freshman class of fall 2011 at the University of Notre Dame, until the spring semester of 2013. Reference^22^ discusses the study’s design and its approval by the university’s Institutional Review Board for studies involving human subjects. The collected dataset includes study participants’ answers to survey questions about their stances on a variety of topics and the Bluetooth proximity records collected continuously by the student’s cell phones. Every phone of a NetSense study member makes a record of every Bluetooth interaction between a pair of participants when this interaction meets the threshold of physical proximity. To extract stable groups formed in each semester from this proximity data, we use the hierarchical clustering method that we proposed in^23^ as discussed in the first subsection of “Methods” section.

Similar data collection strategies and the use of Bluetooth proximity for finding real-world group gatherings were used and validated by others in^8,24–28^. Table 1 lists the details about the groups identified from the Bluetooth data. Our approach discovers 436 groups, with their periods of existence ranging from one to four semesters, and identifies 149,771 meetings. Since the average fraction of group meetings attended by a member ranges from 0.89 to 0.91, members stay with their groups for 90% of the 14 week semester. Accordingly, we assume that changes happen at the three boundaries between the semesters. By design, this is where we analyze any changes in the data. Having so reconstructed groups, we are able to detect changes in these groups and their group members between semesters (see “Methods”). We use these changes as a ground truth for our analytical model, which we design to predict group membership dynamics and changes in individual opinions.

**Table 1:** Properties of groups.

Properties of group structure	Semester			
	1	2	3	4
Number of people in all groups	189	185	171	153
Number of groups	256	269	149	100
Number of meetings per group	177.9	224.5	205.1	132.8
Average number of group members	4.1	3.9	3.9	4.0
Average fraction of attended meetings	0.89	0.91	0.90	0.91

General statistics on the groups detected through hierarchical clustering for each of the available semesters in NetSense.

To study the dynamics of student opinions, specifically, we use demographic surveys that the NetSense study participants completed at the beginning of each semester. These surveys contain 38 questions pertaining to family background, activities on campus, hobbies, and stances on various social and political issues, favorite types of music. The possible answers to these questions also came in a variety of forms, ranging from “yes” and “no” modalities, to closed lists, to open-ended text boxes. In our study, we include the five survey questions that were found to be the most predictive on the formation of new social relationships^29^. These questions are related to general political orientation, as well as stances on the legality of abortion, marijuana usage, gay marriage rights, and alcoholic drinking habits. For political stances, the possible answers rely on a seven-point scale, with one being extremely liberal, four being neutral, and seven being extremely conservative. The other questions use a similar point scale, ranging from strong support to strong opposition to the question, with a neutral stance in the middle. Drinking habits answers describe a range of drinking frequencies, ranging from no drinking to very frequent drinking. For all questions (including drinking habits), we cluster the range of responses into three modalities; joining extreme and moderate stances into two opposing stances while the third stance represents neutral answers. To track a change in opinion across a semester for our ground truth, we simply compare the stances of a student before and after the boundary.

In contrast to other studies that rely on routinely and massively collected Call Record Data, our study relies also on Bluetooth and survey data. This approach allows us to track actual face-to-face groups, rather than hypothetical or ersatz groups purely based on remote communications. While work based on remote telecommunications may have the advantage of scale^30^, it has the disadvantage of not being able to account for face-to-face group dynamics, which is the core scientific question we are after. In this respect, the sample size of human subjects in our study is consistent with that used in the very few other studies that have been able to track on-the-ground human groups over time.

For instance, ^21^tracked 100 participants in an executive MBA program at an elite business school using infrared wearable badges on a single occasion, while^31^ also tracked 100 subjects (using a similar bluetooth technology approach as NetSense) for 9 months. In the same manner, ^20^tracked 24 graduate student participants for a whole year, also using wearable badges. A study that tracked 200 students for a whole year using motion sensors comes close to the sample size in NetSense^19^. All this previous work identifying groups using wearable technology or bluetooth proximity detection is able to identify face-to-face groups using non-obtrusive methods. However, the data used in this previous work lacks measures of people’s opinions, attitudes, and beliefs, and thus is unable to link the observed group dynamics with the opinion distribution across people and groups and related dynamics of opinion change and distribution, as we do in this paper.

The most ambitious effort to study face-to-face groups using computational social science techniques we know of is that of^8^ which, in similar design as NetSense, tracked about 1000 freshmen students from an undisclosed European University for several months determining the existence of groups via bluetooth. However, like the other studies mentioned earlier, ^8^lacks information on individual opinions and beliefs (and thus cannot link group evolution dynamics to opinion change). While smaller than^8^ in terms of sample size, the NetSense data used here has the advantage of tracking participants for almost 2 years, allowing for richer temporal dynamics to be detected. In addition, while^8^ discovers a number of descriptive sociometric dynamics of groups similar to the ones we observe, the authors do not develop an underlying formal model capable of predicting the observed patterns, and thus cannot shed light on the individual micro-mechanisms underlying the observed patterns^32^.

The initial number of participants in the NetSense study is 196. However, its retention rate is $71 \%$ over all four semesters. This is comparable to published studies with similar sample sizes^33^ and avoids the pitfalls of low retention rates which have been shown to seriously affect results^34^. Like previous work, which has studied group dynamics using data collected from “captive” populations (e.g., MBA executives, graduate and undergraduate students, workers at scientific labs and so forth^8,19,20,25^), our study also uses a student population, namely, a sample of Freshmen who began their studies at the University of Notre Dame in 2011. In studying face-to-face groups “in the wild”^20^, it is important to note that the main scientific issue is not necessarily scale or “representativeness” in the probability sample sense. Yet, the University of Notre Dame has a strongly diverse undergraduate student body, both socio-demographically and geographically^22^ with $89\%$ of the students from out-of-state^35^. Thus, we consider these students a representative sample of the life experience for half of the U.S. population born around the year 1993. In that respect, our study is also similar to many other published studies focusing on social network dynamics among individuals undergoing other significant transitions, like going into retirement or having a child^33,36,37^.

Beyond this, studies like ours need to worry about when studying group dynamics in real-life settings is whether the human gatherings detected are in fact face-to-face groups in the sociological sense^1,3^, as the scientific objective is to determine the “life-cycle” of actual groups on the ground, not ersatz groups. Therefore, we need data with high *ecological* validity of the underlying data, study location, and participant population^38^ and the NetSense data provides that feature. Absent high ecological validity, even the largest sample of participants will yield the wrong (or misleading) answers.

University of Notre Dame has a nearly entirely residential campus located on north outskirts of South Bend city, Indiana, in the Midwestern United States. Hence, student life there, especially during the first 2 years of study, revolves mostly around activities (studies, recreation, social and religious life) located exclusively on campus. In that sense, University of Notre Dame is a nearly perfect closed-system, a “social laboratory”, where generic behavioral processes applicable to all human groups can be observed with little exogenous disturbance. For the time of observation, almost all the groups to which individuals belong are located on campus. Additionally, for most of these students, this is their first long-time away from home and family, creating a transformative and shared experience in their social lives^39,40^. The social ties students form at this stage are both personally and sociologically significant as are the groups that they join^1^. Furthermore, at Notre Dame, freshmen students are required to take a specific set of foundational courses heavily oriented toward philosophy, theology, and humanities to delay disciplinary specialization until the second academic year when students can declare majors. This allows for friendships and groups to form invariant of major, as well as keep specialized courses from initially forcing connections between particular students. As such, in this case, given the scientific objective to study general behavioral processes of face-to-face group formation and evolution at fine-grained time-scales, restriction to a student population should pose no barrier to proper inference.

### Sociological processes for determining social group dynamics

Although there is little empirical research on face-to-face group membership dynamics^20^, theoretical work relying on agent-based modeling and simulation in computational social sciences points to three basic processes determining social group dynamics: selective interaction, network-based recruitment, and value homophily. These intuitions can be used as the building blocks to construct a formal model that can shed light on the micro-mechanisms that account for patterns of group evolution observed in the data. First, people selectively concentrate their interactions on members of the groups to which they belong and not on outgroup members^4,41^. The reason is a limited budget of time and energy a person can devote to interactions with others. The more people interact in face-to-face settings, the more likely they are to form strong attachments with one another^1,42^ and to stay with a group^4^. Still, some dyadic ties link members to individuals outside of their current groups^2^. These ties enable individuals to join new groups via the process of network-based recruitment^5,7^. The more time and energy people spend interacting with others outside the group, the more likely it is that they will leave their current groups and join new groups^12^. Hence, the extent to which groups attract an individual’s time and energy is an important determinant of whether individuals will stay with the group^5,7^.

The benefit that people gain from their current group memberships depends on the extent to which they share important attributes with others, referred to as *homophily*. It may include shared socio-demographic characteristics^41,42^, and shared beliefs and opinions, referred to as *value homophily*^41^. People are also likely to leave a group when others disagree with them. Because people share values, beliefs, and opinions via social interaction, selective interaction and homophily can be mutually reinforcing^4^.

### Utility of group member interactions

To account for the aforementioned sociological processes that drive social group dynamics, we develop a formal model based on subjective utility, a common modeling approach in behavioral science^43^. In the model, member *v* gains utility from interactions with member *m* within group *g*. This utility is a function of the interacting members’ fractions of group meeting attendance and their stances on an attribute *a*. As reported in^44^, ruling parties tend to have an equally negative attitude toward members of opposition parties. Thus, we treat holders of neutral and extremist views equivalent in terms of the utility of their interactions with other members of a group. We also assume that the utility gain from interactions with members sharing the same stance is twice as large as the utility loss from interactions with members holding different stances. This assumption yields a simple yet effective model.

In the SM, we show results under an alternative assumption that extremists dislike neutrals less than the opposing extremists while neutrals equally less like interactions with other neutrals. Both models yield similar results. The essential property of both models is that individuals optimizing utility drive up polarization of groups. In the SM, we show that replacing the coefficient 2 in Eq. (1) with a parameter $\alpha \ge 1$ and the coefficient 1/2 in Eq. (S2) with a parameter $\beta \le 1$ preserves this property.

We denote the liberal stance by $-1$, neutral by 0 and conservative by 1. To express relations of stances to each other, we can place them as vertices of the equilateral triangle, with the neutral stance at the top, the liberal stance at left bottom and the conservative stance at right bottom. Then, for each stance *s*, stance $s^+$ denotes its clockwise neighbor, while $s^-$ its counterclockwise neighbor. With this notation, $0^+=1$ and $0^-=-1$, etc. Let for a node *m*, $s_{m,a}$ denotes its stance for attribute *a* while for a member *v* of group *g*, let $w_{v,g}$ denote a fraction of meetings of *g* that *v* attended. Then, utility of *v* from interactions within *g* is

1 $$\begin{aligned} u_{v,g,a} = w_{v,{g}}\left( 2W^{s_{v,a}}_{g,a} - W^{s^+_{v,a}}_{g,a} - W^{s^-_{v,a}}_{g,a}\right) \end{aligned}$$

where $W^s_{g,a} = \sum _{m\in g\cap s_{m,a}=s} w_{m,g}$. Thus, the utility of interactions of node *v* with each member of the group *g*, including *v*, is represented by the product of fractions of the meetings in which each of these two members participated. For each pair of members of the same stance the doubled result is added to the utility, while for each pair with different stances, the result is subtracted from the utility. We include *v* in the list of members of the same stance so it represents the expected vote for this stance. The total utility of node *v* from interactions with members of *g* is just the sum of utility over all attributes. The utility of an entire group is the sum of the total utility of all members of this group.

We hypothesize that the members attempting to maximize their utilities drive group evolution dynamics. Indeed, it is natural to expect that people will seek their own benefit, and that they will make changes in their group membership to seek an improvement to their utility. If this is the only acting criterion when an individual makes a change, this change is *egocentric*. However, participants might also consider the feelings of others when changing group membership or opinions. To account for this, we introduce another criterion based on the average benefits of all group members. This criterion is called *strongly altruistic*, since it is close to the traditional definition of altruism in the social sciences^45^. However, research in economic games also shows that people are more cooperative when they interact with others whom they believe share a group identity^46^ or who share opinions with members of the group^47^. In the dictator game, players in the dictator role transfer more money when they believe the recipient shares their group identity^48^. Hence, it is reasonable to suppose that people making a decision about a change, will take into account how this change will affect members with whom they share a stance on the involved attribute. Accordingly, we refer to a change as *weakly altruistic* if the total utility change for all members who share the most of their stances, and thus, value homophily, with the person making the change, is positive.

Formally, all three types of changes use the same function computing a difference in utility for a certain subset of members of a group *g* on which node *v*, making a change, focuses on. This subset contains just *v* for egocentric change, the entire group for strongly altruistic change, and all nodes in *g* with the same stances as *v* for weakly altruistic change. Therefore for node *v* in a group *g*, we compute the utility change by subtracting *v*’s utility before the change from this utility after the change. A change is accepted if, across all attributes, the sum of utility changes for all member in the focused subset is positive.

**Table 2:** Dynamics of altruism and group polarization.

Semester	Percentage of changes that are			Group polarization	
Boundary	Egocentric	Altruistic		Percentage	Absolute
		Weakly	Strongly	Increase	Difference
1-2	90.6	80.4	76.6	64.0	2.64
2-3	92.4	84.0	81.4	49.1	1.96
3-4	95.3	89.2	86.2	37.4	1.80

Analysis of the total number of altruistic changes and the growth of group polarization across the three semester boundaries.

We computed the frequency of the three types of criteria in the empirical data. The left side of Table 2 shows the results. On average, $93 \%$ of changes made by members are egocentric, $85 \%$ of them are weakly altruistic, and $81 \%$ are strongly altruistic. These results show that large majority of individuals make group changes benefiting not only themselves, but also others. It seems counter-intuitive that an average of $7 \%$ of changes result in no utility benefits for the change-making individual in any way. Yet, these types of changes are likely made due to sudden and unexpected circumstances that cannot be traced by our indicators of value homophily. E.g., leaving a group due to the discovery of irrevocable differences of personalities with some members). Interestingly, $81\%$ of changes also benefited those who did not even align their stances with the node making a change. This might well be an unintentional consequence of egocentric changes. For example, when a holder of minority stances in a group leaves motivated by the low utility this holder is gaining, all holders of majority stances gain utility, and their total gain may be higher than the utility lost by the peers of the leaving member.

Additionally, we found that the differences in utility resulting from the changes made versus not made in the data are always positive. Yet, their magnitude depends on the type of changes compared (see “Methods” for details). The biggest differences arise for egocentric changes. Yet, they vary significantly from 27 to $45\%$. The smallest are seen for the strongly altruistic changes, for which the differences vary the most from 2 to $32\%$.

In addition, the utility gains observed in each semester increase group value homophily. This increases stance polarization across groups. To quantify this change, we define a measure of polarization as a function of stance alignment. To measure polarization across a group, we sum the squares of differences between globally expected fractions of stances of all attributes and the actual fraction of these stances in each group. More formally, let $W^s_{g,a}$ denote the sum of fractions of attendances for all group members in *g* with stance *s*, while $W_{g,a}=\sum _{s={-1}}^1 W^s_{g,a}$ denote the sum of such fractions for all group members. Then, $G^s_{g,a}=w_{g,a}\frac{\sum _g W^s_{g,a}}{\sum _g W_{g,a}}$ denotes the expected attendance of stance *s* for attribute *a* in group *g*. With this notation, the polarization of group *g* on attribute *a* can be expressed as

2 $$\begin{aligned} P_{g,a}=\sum _{s=-1}^1 \left( w_{g,a}G^s_{g,a}-W^s_{g,a}\right) ^2. \end{aligned}$$

**Figure 1:**
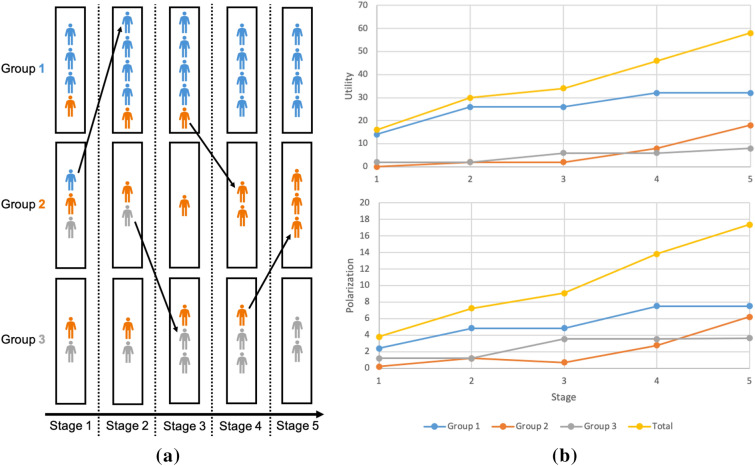
Example of group dynamics with polarization and utility growth over time. (**a**) Evolution of group memberships, with three potential groups and nine different participants. These individuals hold three different stances (each marked by its own color, either blue, orange, or grey) for some arbitrary attribute. (**b**) The increase of group utility and group polarization in discrete steps as the members of the example groups change membership to maximize their utility. All steps made are egocentric and altruistic.

The total group polarization is just the sum of the values of this function for each attribute. Polarization is 0 when there is a full agreement between the global and local frequencies of stances among members. It grows when members increasingly align their stances with each other. This can be accomplished within each group by members either changing their stances or leaving groups in which their stance is the minority, and joining those where their stance is the majority. Figure 1 illustrates a group evolution in which in each step both utility and polarization increase for groups involved in a change.

For each semester boundary, we use our polarization measure to compute the actual trend of value homophily within the NetSense data by subtracting polarization for each group before a change from this polarization after the change in membership. The right side of Table 2 shows the results that show polarization monotonically increasing over time.

Now, by examining Eq. (1) for utility and Eq. (2) for polarization, it is clear that increasing group homophily imposes stronger polarization and a sequence of maximal (i.e., increasing utility the most) strongly altruistic changes irreversibly push groups toward the full stance polarization^49^. Given this, we can conclude that for any state in a group not fully homogeneous, there is a sequence of strongly altruistic changes leading toward complete polarization. These steps increase consensus on stances among group members, showing that value homophily has strong effects on the society-wide distribution of stances. Since $81\%$ of all changes in the NetSense data are actually strongly altruistic, the above trends are strong in this empirical dataset.

Analyzing the NetSense data even further, we also discover that students holding the campus majority stances change their group membership less frequently than do those holding less popular stances. The data shows that quantitatively, the majority stance holders moving to the next semester retain membership in about $85\%$ of the groups while such fraction for students with the minority stance is $75\%$. Additionally, we find that the majority stance holders not only retain membership of groups for a greater amount of time, but they also enjoy a majority control of membership in a greater number of groups. We find that on average, the number of groups in which the majority of members hold the minority opinion is just 53%, barely over half of the number of groups with a majority of members holding the majority opinion. We also measure the dependence of utility gained on the popularity of stances of a group member on four attributes (we excluded the drinking habits attribute since its values represent ranges of frequency, not stances). The ratio of the average utility gained by members with the minority stance to such utility earned by members with the majority stance is the highest for the legalization of gay marriage (0.55), followed by political leaning (0.29), legalization of marijuana (0.25), and abortion (0.11). We also present the fractions of people with majority and minority stances for the entire population and for the top $10\%$ of members, ordered by the utility they received. Table 3 presents these results.

**Table 3:** Majority and minority stance analysis.

Fractions of:	All individuals with		High utility individuals with	
	Majority stance	Minority stance	Majority stance	Minority stance
Politics	0.55	0.16	0.74	0.10
**Legalization of**				
Marijuana	0.54	0.14	0.75	0.03
Abortion	0.49	0.13	0.63	0.13
Gay marriage	0.54	0.20	0.68	0.12

A comparison between stance popularity for fractions of nodes with majority and minority stances among all members and among the top $10 \%$ of members with the highest utility.

We find that the nodes gaining high utility are more likely to hold majority stances than expected by chance based on their fraction in the entire population. Likewise, the high utility nodes are statistically less likely to hold a minority stance, as indicated by the fraction of minority stance holders in the entire population.

To summarize, this section demonstrates that our utility increases for $93 \%$ of the changes made by members in the NetSense dataset and $81 \%$ of all changes also increase utility across the entire group. Moreover, nodes observed to make frequent group membership changes have lower utility than other nodes.

This validates our hypothesis that members attempting to increase their utilities are driving social group dynamics.

### Predicting group membership and opinion changes

To test this hypothesis further, we implement an analytical model to forecast future group affiliation and stance changes based on this hypothesis. We hypothesize that maximizing a person’s total utility from group memberships also requires accounting for the time and effort of attending group meetings. Therefore, the total utility for predictions is then the sum of two terms: the ingroup interaction utility and the utility of membership in the given number of groups. Our model analytically computes the ratio of these two terms that maximizes the prediction performance on training subset of our data (the first two semester boundaries), and then predicts membership and opinion changes that specifically improve utility on the remainder (the last semester boundary). To give some reference of the quality of performance of our model, we use a random baseline that randomly predicts changes across our data (see “Methods” for details on the analytical model, and SM for the baseline implementation).

**Table 4:** Performance of the models.

Measure	Group joining	Group leaving	Stance change
Precision	88% (0.28%)	26% (2.8%)	58% (21%)
Recall	84% (0.23%)	93% (2.8%)	50% (27%)
F1	86% (0.24%)	41% (2.7%)	54% (22%)

A comparison of the Precision, Recall, and F1 between the analytical model and the random baseline model. The baseline model’s results are included in the parentheses.

Table 4 shows the results for the test data predictions of both the analytical model and the random baseline compared to our ground truth. The results indicate that group joining behavior is more predictable than both group leaving and stance change behavior. This is important, as the random model shows that, as a baseline, this task is very difficult to perform. This is because for group leaving and opinion changes, a person considering a change belongs only to a few groups to leave and holds a few opinions to change. On the other hand, there many groups that are open and available to join for such a person. Still, even for group leaving and opinion changing, prediction quality of the analytical model is substantial when compared to the baseline. Overall, the results demonstrate that the analytical model is very effective at predicting changes. These results also validate maximizing the group membership utility as a process driving group dynamics.

## Discussion

Previous theoretical and simulation-based works in computational social sciences and complex networks has pointed to a variety of processes involved in selecting group affiliation by humans. Those include network ties, rates of interaction and time investment, and shared knowledge and opinions^2,4,5,7^. Yet, empirical validation of these processes and their relevance to explaining and predicting group affiliation and opinion change behavior has been scant. The main reason is a paucity of naturalistic data in which there is a mapping from such behavior to data on beliefs and opinions of group members.

In this paper, we are using the NetSense study dataset, which is unique in containing longitudinal information about communications, locations, and opinions for randomly selected students from the University of Notre Dame geographically diverse student body. We detected in this data 436 groups and their evolution across four semesters of activities. The data captures a large representative sample of social group dynamics within the context of college life. We observe that groups slowly transition toward ingroup stance homogeneity. Moreover, we find that the frequencies of group changes by individuals strongly depend on popularity of their stances. We use these empirical observations to formulate and validate our hypothesis about the group evolution.

Theoretical works have postulated that the level of homophily with other members in groups and communities defines the benefit of membership^4,7^. Recent works have also analyzed the roles of homophily in members popularity^15^, ranking of minorities^16^, and structural change^17^, respectively.

In this paper, we formalize a notion of utility of group membership as a function of compatibility of opinions between a member and all others in its group. This formalization enables us to integrate in a single model the three aforementioned phenomena and to account for the two dynamic patterns observed in empirical data. Using this data, we simulate only those changes of opinions or group memberships, which increase members’ utility. We find that this process recreates the group dynamics observed in empirical data and confirms the theoretically postulated role of homophily in these dynamics.

The total member utility includes another term, which is a function of the number of groups to which this member belongs to account for commitment limit each person has. We demonstrate that when applied to our empirical data, this utility increases after each observed change. This leaves an interesting open question: whose average utility should a member maximize when considering a change of an opinion or group membership? To probe different answers, we introduce three maximization criteria. The first, called egocentric, requires that the change of utility of a decision-maker needs to be positive for the move to be accepted. We also consider a more considerate alternative in the form of the weakly altruistic criterion, which requires the positive average change of utility for group members sharing stances with the node making the change. Finally, we produce a strongly altruistic criterion, when all group members on average benefit from a change.

We find that, on average, $93 \%$ of changes made by students in NetSense data are egocentric and $85 \%$ are weakly altruistic. Additionally, over $76 \%$ of these changes are also strongly altruistic at the first semester boundary, with this fraction growing to $86 \%$ at the third semester boundary. These changes increase polarization, resulting in its observed growth. These results empirically confirm that it is important to allow for an altruistic component in agreement with previous work linking altruism, empathy, and group identity. Another observation from empirical data finds the differences in frequency of group membership changes between individuals espousing majority and minority stances. Our utility decays with the decreasing popularity of the member stances. This naturally increases such member motivation to attempt changes to maximize the utility. This agreement with empirical observations validates our hypothesis that utility maximization drives group dynamics.

Subsequently, we introduce a predictive analytical model based on utility maximization to forecast the evolution of groups. It balances the two terms of total utility at the value that globally maximizes model performance on training data. This model accurately predicts affiliation (group joining and group leaving) and stance change.

Overall, these results advance our understanding of group dynamics. They also have important implications for future work on this topic in social sciences, computational social systems and complex networks. In particular, our results show that core processes isolated in previous theoretical and simulation-based work are applicable to naturalistic settings, uncovering the motivations leading people to join or leave groups. We also identify two side effects of utility maximization. The first is that holders of unpopular stances gain lower utility from ingroup interactions and have increased frequency of group changes. The second reveals that desire to spend more time interacting with like-minded others contributes to the increased stance polarization across groups. This kind of polarization has been noted previously^50^, where seemingly innocuous stances and beliefs become highly influential markers determining who interacts with whom, generating small “echo chambers” characterized by opinion homogeneity. This is especially relevant in contemporary contexts featuring relatively low barriers to geographic mobility allowing persons to self-select into social environments and to find and affiliate with groups of their choice online or face-to-face.

## Methods

### Group detection within NetSense

The first step in analyzing group dynamics within the NetSense dataset is to extract groups from the Bluetooth proximity data. As mentioned in the main text, we extract groups from this proximity data using the hierarchical clustering method proposed in^23^. This method first finds persistent connected components in the dynamic network generated from NetSense’s dyadic Bluetooth interactions. Each detected component is a potential group meeting. The largest sequence of components such that each component includes at least a fraction $f_i$ of the union of the members of all other components is considered a representation of a meeting of a single group. Members of this group are the nodes that attend at least a fraction $f_m$ of the meetings of this group. Finally, each meeting that attracts less than a fraction $f_{mi}$ of its group members is removed. The details of the algorithm to extract groups with the required properties and for finding the best values of parameters that are $f_i=0.6, f_m=0.5, f_{mi}=0.3$ are presented in^23^. Since this reference uses the same NetSense data as our study does, we use these parameter values to extract groups.

Bluetooth interactions collected on the phones of participants include proximity data of all cell phones. Yet, we found that the ratio of non-participant meetings with participants (required to establish a group) to participant meetings with participants is 0.0489. Thus, no phone owned by a person not enrolled in the NetSense study passed the described above group membership requirements. As the result, the extracted groups include only participants of the NetSense study.

Since groups are not labeled in the data we work with, we must extract a mapping to identify a self-subsisting group across consecutive semesters. The mapping uses the Jaccard Similarity on the two sets of members of both groups. A threshold is set for similarity level for the latter group to be a continuation of the former. By tracking these mappings across semesters, we can identify new or missing members between subsequent group reincarnations. We consider so-identified new members as the true positive cases for joining the affected group and treat missing members as ground truth cases for leaving. However, when an entire group finds no succeeding group mapping in the following semester, we do not record the ingroup participants as a ground truth case of leaving the group en masse. Instead, we just dissolve the group by removing each node from this group for the new semester. The reasoning behind this decision is that the data collection process for NetSense could sometimes be noisy^22^ causing the matches to be lost.

With these consistent initial group mappings, we observe in the ground truth data that the majority of people join groups with members with whom they share some communication links. This is in agreement with sociological work on network-based recruitment into groups^5,7^ discussed previously. To account for this phenomenon, we introduce a lower bound on the number of connections that a given person has with current members of the new group over all meetings in which they participate. This bound is the product of the fraction of group’s members linked via communication contacts to that individual in the current semester and the number of meetings held by the group’s members, which measures group stability. We selected the bound value in such a way that a typical group with five members of which two are familiar to the person attempting to join would require holding 50 meetings to qualify. However, just ten meetings would have sufficed if the joining person had connections to all five members.

### Utility difference for changes

Given group mappings between semesters, we can track for each participant all the changes made and not made in group memberships. For example, every group to which participant *v* does not belong in the subsequent semester is a group that *v* could have joined, but did not. Additionally, every group that *v* is a part of in the following semester is a group that *v* could have left, but abstained from doing so. Between the 196 NetSense participants, 889 group membership and opinion changes were made across all three semester boundaries, and 16,348 possible changes were not. To quantify the actual utility differences between changes made and changes not made, we subtract utility of node *v* in group *g* for some attribute *a* before the change is made from this utility after the change. For a node joining a group without an associated attendance history, the average frequency of attending meetings of its other groups is used, and the frequency is set to 1 if the former is not available. For opinion changes, the total utility change is computed by subtracting the sum of the utility for each group to which node *v* belongs before the change from that sum after the change.

### The analytical predictive model

Our model aims to predict the set of changes maximizing the utility individuals derive from the groups to which they belong or will join with the opinions they hold. In this analytical model, the utility functions use a model parameter *x*. It defines an exchange rate between utility derived from ingroup interactions versus an adjustment representing the commitment of belonging to the current number of groups. Our analytical model maximizes this augmented version of utility. To find the optimal *x*, we define a penalty function for changes predicted by the model but not made in reality (false positive changes) and for changes not predicted by the model but made in reality (false negative). There is no penalty for changes predicted correctly (true positive) or changes not made and predicted as such (true negative). Using training data and this penalty system, we find the optimal value of the parameter *x*. The exact mechanism for analytically solving for the optimal *x* is shown in the next subsection. Overall, to predict change in group membership and opinions, the model finds the augmented utility differential by subtracting utility before a simulated change is made from this utility after the change.

Equation (1) formally defines the augmented utility for a node *v* in group *g* with a stance on attribute *a*. Summing over all attributes and over all groups to which *v* belongs defines the overall utility derived by node *v* from all ingroup interactions. However, belonging to a group requires the commitment of time, a resource limited for all humans, for attending meetings. Human interactions satisfy a genuine human need, but this part of utility decays from the utility with the optimal time commitment to groups for both under and over participation in groups. This decay grows non-linearly with the imbalance of the committed time. To account for this, we define a quadratic function for each node *v*, $f^g(v)=W_v(2{\overline{W}} - W_v)$, where $W_v=\sum _{\{g|v\in G\}} w_{v,g}$ is the total time commitment of node *v* to all groups to which this node belongs and *G* denotes a set of all currently existing groups. ${\overline{W}} = \frac{1}{n} \sum _{v \in P} W_v$ is the average time commitment of all nodes to all groups where *P* denotes a set of all participants, and *n* stands for the total number of nodes. Using the data from the first two semester boundaries to establish an empirical value of average, we found that ${\overline{W}} = 3.95$. The sum of the ingroup interaction and time commitment utility of a node represents its augmented utility.

Both terms of this augmented utility are function of time, but represent different units, therefore we introduce an exchange rate, *x*, between the two. Hence, a person *v* who belongs to group *g* and holds a set *A* of opinions (attribute values) in a given semester, gains the augmented utility defined as

3 $$\begin{aligned} f^u_v = x f^g(v) + \sum _{g \in G_v} \sum _{a\in A} u_{v,g,a}, \end{aligned}$$

where $G_v$ denotes the set of groups to which node *v* belongs. When considering a membership change, our model evaluates Eq. (3) before and after the change is made, subtracting the former from the latter. If the difference is positive, the move is eligible for execution, otherwise it is not. For opinion change, the model subtracts the sum of augmented utilities for *v* in all the groups to which *v* belongs before a change from such utility after the change.

The non-linearity of the commitment function makes the eligibility of moves dependent on the order in which they are attempted. When the current number of groups to which a node belongs is below four, the new group joining proceeds to trial before any other change. Otherwise, the group leaving, if one exists, has the execution priority. Opinion changes proceed only after all other changes have finished their trials.

### Analytically defining the optimal *x*

To find an optimal value of *x*, we define penalty functions for group affiliation and opinion changes. Our model minimizes the total penalty incurred for all individuals within our training data (the first two semester boundaries) to find the globally optimal value of *x*. The total penalty used is

4 $$\begin{aligned} C = \min _x \sum _{v \in P} \sum _{t=1}^{S} \left( \sum _{g \in G^{-}_{v}} p^l_{g,v,t}+\sum _{g \in G^+_v} p^j_{g,v,t}\right) , \end{aligned}$$

where *C* represents the total penalty for all individuals considering a change, *x* denotes the exchange rate parameter, *v* is the change-making individual, *t* represents the semester, *P* is the set of all study participants, and *S* is the constant denoting the number of semesters. $G^{-}_{v}$ denotes the groups of which *v* is currently a member, and $G^+_v$ denotes the groups that *v* is eligible to join in semester *t*. Value $p^l_{g,v,t}$ is the penalty for leaving or not leaving group *g* in semester $t+1$, and $p^j_{g,v,t}$ is the penalty for joining or not joining the group *g* by *v* in semester $t+1$.

#### Leaving a group

We can construct the leaving group penalty function based on the utility function as follows:

5 $$\begin{aligned} p^l_{g,v,t} = |\Delta ^l_g f^u_v|-m^l_{v,t}\Delta ^l_g f^u_v, \end{aligned}$$

where $\Delta ^l_g f^u_v$ is computed by subtracting the augmented utility $f^u_v$ before *v* leaves group *g* from that utility after the leave, while $|\Delta ^l_g f^u_v|$ denotes the absolute value of this difference. Using the absolute value in the term containing the unknown parameter *x* makes the entire function non-linear with respect to *x*. The factor $m^l_{v,g,t}$ represents if person *v* leaves group *g* in semester *t*. In particular, if this person indeed makes the predicted change in the ground truth data, then the value of $m^l_{v,g,t}$ is 1, otherwise, it is $-1$. The intuition behind such a penalty function is that a person avoids the penalty for a change if and only if the resulting utility differential is positive.

For changes with a positive utility, the value of $m^l_{v,t}$ is equal to 1, so no penalty is incurred when the change is positive. However, if the value of $m^l_{v,t}$ is equal to $-1$, a penalty is incurred by the model for predicting a change not made in the ground truth data. Conversely, a change associated with a negative utility differential, but made in the ground truth data incurs a penalty since with $m^l_{v,t}=1$, the absolute value of the second term is added to the first. In opposite case, when the second term is equal to the first and when $m^l_{v,t}=-1$, the expression in Eq. (5) reduces to 0.

#### Joining a group

We construct the joining group penalty function by replacing the difference in the augmented utility for leaving a group in Eq. (5) with the utility differential of joining a group from. Hence, the penalty for joining a group, $p^j_{g,v,t}$, has properties analogous to properties of penalty for leaving a group. Naturally, the weight of node *v* is not known before the individual joins the group. Therefore, as before, we use either the average rate of all of *v*’s other groups or set the attendance rate to 1.

### Solving for the optimal *x*

The optimal value of *x* defined by Eq. (4) is efficiently solvable by replacing each derivative of an absolute value of a term with the product of the sign function of this term and the derivative of this term, yielding the expressions in the form

6 $$\begin{aligned} \frac{p^l_{g,v,t}}{dx} = \text {sign}\left( xA^l_{g,v,t} - B^l_{v,t}\right) (1-m^l_{v,t}) A^l_{g,v,t}, \end{aligned}$$

where $A^l_{g,v,t}=w_{g,v}(2W_v-w_{g,v}-2\overline{W})$ and $B^l_{v,t}=\sum _{a\in A}2W^{s_{v,a}}_{g,a}-W^{s^+_{v,a}}_{g,a}-W^{s^-_{v,a}}_{g,a}$, where *g* is a group that node *v* is leaving. Each term defined by Eq. (6) changes its value only once for $x=B^l_{v,t}/A^l_{g,v,t}$. We will refer to these values as signum discontinuity points. The product of the number of nodes and the number of groups whose memberships are subject to change is the upper bound for the number of signum discontinuity points. After sorting these discontinuity points from smallest to largest, and processing them in this order, we can than find optimal value of *x*, by computing the derivative’s value at each discontinuity point to identity the smallest value of penalty and corresponding value of *x* in the current interval between the previous and the current discontinuity point. After processing all discontinuity points, we will have the minimum penalty and the value of *x* at which it is reached. For our data specifically, using the observations from the first two semester boundaries, we found the optimal *x* to be $1.4095 \times 10^{-4}$.

## Supplementary Information

Supplementary Information 1.
