# Supplementary material for: Creation, evolution, and dissolution of social groups
Source: Sci Rep. 2021 Sep 1;11:17470. doi: 10.1038/s41598-021-96805-7 (PMC8410948; doi:10.1038/s41598-021-96805-7)
Supplement: Supplementary file 1 — Supplementary Information 1. [file 41598_2021_96805_MOESM1_ESM.pdf]

Supplementary Materials for
Creation, Evolution, and Dissolution of Groups in Social Networks

James Flamino, Boleslaw K. Szymanski, Ashwin Bahulkar, Kevin Chan, Omar Lizardo

Methods

Asymmetric Utility

In the manuscript, the formal definitions for the individual utility functions are "symmetric", in the sense that all stances for each attribute have the same impact on other stances. Therefore, the resultant deltas used to interpret the altruistic criterion are also symmetric. However, an alternative portrayal of utility as a function of ingroup opinion alignment is also valid. In it, group members with a neutral stance on a are only half as potent as their counterparts with non-neutral stances. This implies that neutral members of the group do not affect the balance of utility within g as greatly as the non-neutral members do.

In the following, we continue to use the notation defined in the manuscript. Hence $w_{v,g}$ denotes the fraction of attendance of node v in the meetings of group g, while $s_{v,a}$, $s^{+}_{v,a}$, and $s^{-}_{v,a}$ denote the stances of node v and its clockwise and counterclockwise neighbors for attribute a, while $W^{s}_{g,a}$ denotes the sum of weights of nodes in group g with stance s on attribute a. To abbreviate notation, let $M^{s}_{g,a} = \frac{1+|s|}{2} W^{s}_{g,a}$, so for neutral stance $s = 0$, it is $\frac{W^{s}_{g,a}}{2}$ and just $W^{s}_{g,a}$ otherwise. Given these definitions, we define asymmetric utility as

$$u_{v,g,a} = w_{v,g} \left(M^{s_{v,a}}_{g,a} - M^{s^{-}_{v,a}}_{g,a} - M^{s^{+}_{v,a}}_{g,a} \right). \tag{1}$$

Hence interactions involving at least one neutral use the coefficient $\frac{1}{2}$, otherwise the coefficient is 1. The resulting utility is positive when both nodes agree on the stance for the corresponding attribute, and negative otherwise. Formally, the group utility function for attribute a, denoted as U^{a}_{g}, can be expressed as

$$U^{a}_{g} = \left(W^{1}_{g,a} - W^{-1}_{g,a} \right)^2 + W^{0}_{g,a} \left(\frac{W^{0}_{g,a}}{2} - W^{1}_{g,a} - W^{-1}_{g,a} \right). \tag{2}$$

Now, we can represent the deltas for leaving and joining group v with stance s on attribute a under weakly and strongly altruistic conditions as

$$\Delta^{r,s_{v,a}}_{g,v,a} = M^{s_{v,a}}_{g,a} - M^{s^{+}_{v,a}}_{g,a} - M^{s^{-}_{v,a}}_{g,a} + \frac{1+|s_{v,a}|}{2} w_{g,a}, \tag{3}$$

where r is equal to $+1$ for joining a group and -1 for leaving it for weakly altruistic changes, and twice as much for strongly altruistic changes. Given these, the condition for a move to be weakly altruistic is to evaluate the above equation, summed across all attributes. If the result is positive, then the move is altruistic. For the egocentric moves, we assign a utility 0 to node v when they are out-of-group, calculating asymmetric utility otherwise, then finding the difference (which must be positive to be egocentric).

Table S1. Changes with asymmetric altruism. Fraction of total changes under the altruistic and egocentric criteria with the asymmetric altruism method, evaluated across the three semester boundaries.

Semester Boundary	Fraction of Weakly Altruistic	Fraction of Strongly Altruistic	Fraction of Egocentric
1-2	0.95	0.76	0.76
2-3	0.93	0.82	0.82
3-4	0.88	0.69	0.69

Using the alternative altruism and egocentric conditions defined in the previous section, we can analyze the altruistic tendencies of the person making changes in the three semester boundaries from a different perspective. Here, we discovered that the extended definition of the neutral stance increases variation of the results. However, Table S1 shows that, on average 92% of moves made per semester boundary are weakly altruistic, while on average 76% of such moves are also strongly altruistic.

Additionally, 76% of moves were also egocentric. Ultimately, the results are strong enough to indicate that there is a propensity for altruism in ingroup behavior. Yet, it is weaker here than under the symmetric altruism.

To compare these two models on their shifts toward opinion homogeneity in affected groups, we subtract the group utilities before a change from this utility after the change. We also find that 96% of weakly altruistic changes resulted in opinion segregation. The percentage of polarizing changes is still very high, confirming the presence of polarization shift under this regime. Given the great overlap between the results of asymmetric and symmetric altruism models, we conclude that there is a clear trend toward opinion homophily. Ultimately, the two interpretations of altruism presented here show only small differences in the dynamics of group affiliations. We observed some noticeable differences only in distribution of utility in interactions among subgroups with different alignments of opinions. The resultant equations comprehensively preserved the properties of group dynamics (which we found to be evolving toward a polarized stable state).

The Generalized Models

Here, we show that the essential property of our models that the utility optimization by individuals results in group polarization is not dependent on the specific values of utilities assigned to interactions of members of a group. In the case of the symmetric utility model introduced in the manuscript, we can replace the constant 2 with a parameter $\alpha > 1$ defining the ratio of utility gain from interactions of a pair of members with same stance to the loss from interaction of pair members with different stances. Thus, the corresponding equation becomes

$$U_g^a = \alpha \left[\left(W_{g,a}^1 - W_{g,a}^{-1} \right)^2 + \left(W_{g,a}^0 \right)^2 \right] - 2 \left(W_{g,a}^0 + \alpha - 1 \right) \left(W_{g,a}^1 + W_{g,a}^{-1} \right). \quad (4)$$

Representing the deltas for leaving and joining group v with stances on attribute a under weakly and strongly altruistic conditions yields inequalities similar to those shown in Eq. S3. Interestingly, the larger the value of α, the faster the system convergence to fully polarized groups.

Likewise, for the asymmetric utility model, we can replace the constant $1/2$ with a parameter $0 < \beta \leq 1$ defining utility ratios of interactions of pairs of members involving members with neutral stances. The corresponding equation is

$$U_g^a = \left(W_{g,a}^1 - W_{g,a}^{-1} \right)^2 + \beta \left[\left(W_{g,a}^0 \right)^2 - 2 W_{g,a}^0 \left(W_{g,a}^1 + W_{g,a}^{-1} \right) \right]. \quad (5)$$

Again, the deltas for leaving and joining group v with stances on attribute a under weakly and strongly altruistic conditions yield inequalities similar to those based on Eq. S4. Thus, the model has similar dynamics as the earlier discussed models. However, in this case, the paths to polarization differ slightly between systems with values of the parameter β that are close to its range boundaries (i.e. 0 and 1).

The Random Baseline

To provide a practical comparison against our analytical model, we introduce a simple random model to act as a baseline for our group membership and opinion change predictions. To account for the first two semesters of training data, we model some prediction thresholds using that data to dictate how students will be selected to make random moves for the test data. First we find how many students were active in the testing data for semester boundary i: n_i. We then find the number of students belonged to groups at semester boundary i and denote it as ng_i. Given these, we find the number of students that left at least x_l groups in the first and second semester boundary denoted as $nl_1(x_l)$ and $nl_2(x_l)$, respectively, where x_l is the minimum number of groups left. From this we can craft the threshold $pl(x_l) = (\frac{nl_1(x_l)}{ng_1} + \frac{nl_2(x_l)}{ng_2})/2$. We compute the same thresholds for group joining and opinion change, yielding the thresholds $pj(x_j)$ and $pc(x_c)$ where x_j is the minimum number of groups joined and x_c is the minimum number of opinions changes. We also define a threshold for the number of students that were not initially part of a group that subsequently joined a group by finding the number of non-members that joined at least x_{nj}groups for each semester boundary denoted as $njn_1(x_{nj})$ and $njn_2(x_{nj})$. With this notation, we express the threshold as $pjn(x_{nj}) = (\frac{njn_1(x_{nj})}{n_1 - ng_1} + \frac{njn_2(x_{nj})}{n_2 - ng_2})/2$. Since these thresholds were learned from the training data, we begin to iterate through all active students to make random membership and opinion changes to compare against the test data.

For each active student, we check to see if that student was part of any groups in the previous semester before the test data. If this is true, we generate a sequence of random numbers to compare against each of the first three thresholds. Each threshold is evaluated independently, so any considered student can make all three possible types of changes if they are lucky enough. For group leaving by some arbitrary active student, we find select a random number r, where $\{r \in \mathbb{R} | 0 < r < 1\}$, We then check if there is index l such that $r < pl(x_l)$. If there is one, then the student will randomly leave a group, which is randomly chosen from the set of groups to which this student belongs (no selection replacement). Otherwise, the student keeps all his current memberships. This entire process is then done for group joining, and opinion change. If the currently considered active student was not a member of any groups in the past semester, we simply repeat the above process for the $pjn(x_{nj})$ thresholds. We also evaluate for this student opinion change, but not group leaving as there are no groups to leave.

This model was run and compared against the ground truth $N = 1000$ times. The presented results average over these iterations. Table 4 in the manuscript shows that the random model performs poorly in terms of change prediction when compared to the ground truth. It performs especially poorly for group joining, as there are many groups to choose from (especially considering the small number of groups to which the average student belongs). Using Eq. 1 and Eq. 2, we compute the group utility and group polarization changes by subtracting utility and polarization before random choices are made from the after those changes. We find that, for the test data, there is an average 4.10% decrease in group utility and an average 4.46% decrease in group polarization. These results, compared to the actual trends seen in Table 2 in the main text, indicate that random moves ultimately decrease the shift toward opinion homogeneity. This makes sense, as random choice does not account for the underlying mechanic of utility maximization, so a majority of moves will find students ending up arbitrarily in groups that are not aligned with them at all in terms of stances.
